# Does cleaning of post space before cementation of fiber reinforced post affect the push-out bond strength to resin cement?

**DOI:** 10.1186/s12903-025-07483-0

**Published:** 2025-12-22

**Authors:** Maher S. Hajjaj, Khalid A. Alghamdi, Abdulrahman A. Alshehri, Hassan A. Almusallam, Nabeel M. Munshi, Osamah A. Alsulimani, Naseeba H. Khouja, Yousef A. Alnowailaty, Saeed J. Alzahrani

**Affiliations:** 1 Department of Restorative Dentistry, Faculty of Dentistry, King Abdulaziz University, Jeddah, Saudi Arabia; 2 Family Dentistry Department, Riyadh Elm University, Riyadh, Saudi Arabia; 3 Advanced Education in General Dentistry Department, King Abdulaziz Medical City for National Guard, Riyadh, Saudi Arabia; 4 Clinical Instructor, Faculty of Dentistry, King Faisal University, Hufof, Saudi Arabia; 5 Department of Oral and Maxillofacial Prosthodontics, Faculty of Dentistry, King Abdulaziz University, Jeddah, Saudi Arabia; 6 Department of Oral Diagnostic Sciences, Faculty of Dentistry, King Abdulaziz University, Jeddah, Saudi Arabia

**Keywords:** Root Canal System, Decontamination, Bond Strength, Post and Core, Cleaning Post Space, Bonding

## Abstract

**Background:**

Fiber-reinforced posts (FRPs) are preferred for the rehabilitation of endodontically treated teeth. Achieving adequate bond strength between resin cement and root dentin requires effective removal of the smear layer and debris from the post space before cementation. The aim of this study is to evaluate the effect of various post space cleaning methods prior to cementation of fiber-reinforced posts on the push-out bond strength (PBS).

**Methods:**

Ninety-one human premolars underwent root canal treatment, post space preparation and were assigned to seven groups, each using a different cleaning method. These included normal saline (control), sodium hypochlorite, chlorhexidine, EDTA, air abrasion, Katana™ Cleaner, and phosphoric acid. Tapered fiber-reinforced posts were cemented using self-adhesive resin cement. Two middle root slices from each tooth were obtained (26 specimens/group). Then, all specimens were subjected to thermocycling followed by push-out testing. Failure modes were evaluated under a stereomicroscope. Statistical analysis for PBS was performed using Kruskal-Wallis test and Chi-square test was used to analyze failure modes.

**Results:**

The mean PBS ± Standard deviation of all groups, ranked from highest to lowest were; Phosphoric Acid 26.61 ± 7.22 MPa, Chlorohexidine 21.33 ± 5.54 MPa, Sodium Hypochlorite 17.77 ± 4.37 MPa, EDTA 16.76 ± 4.54 MPa, air-born particle abrasion 15.72 ± 4.61 MPa, Katana^™^ Cleaner 15.48 ± 4.06 MPa, and Control 12.73 ± 3.53 MPa. The control group exhibited the lowest PBS, and only phosphoric acid, Chlorohexidine and Sodium Hypochlorite demonstrated significantly higher PBS than control group. A higher frequency of favorable failure mode (Failure Mode 1) was seen in groups treated with phosphoric acid (80%), chlorhexidine (90%), and EDTA (80%), all of which demonstrated higher push-out bond strengths.

**Conclusions:**

Post space cleaning significantly affects PBS. Failure to properly clean the post space before cementation negatively affects the post retention. Cleaning with phosphoric acid demonstrated the highest push-out bond strength, more than double that of control group, followed by chlorhexidine and sodium hypochlorite.

**Supplementary Information:**

The online version contains supplementary material available at 10.1186/s12903-025-07483-0.

## Background

Endodontically treated teeth with extensive loss of coronal tooth structure often require post and core restorations to provide sufficient retention and stability for the final restoration [1]. This coronal loss of tooth structure influences the resistance of the tooth to the fracture during function [2]. These restorations serve as a foundation for prosthetic crowns, ensuring both functional and esthetic outcomes. There are two main factors that influence the clinician’s decision to select the technique to restore the endodontically treated tooth: the tooth location and the remaining tooth structure [3]. Restoring missing tooth structure with a core build-up might make the indirect restoration more susceptible to dislodgement forces, potentially affecting its durability [4]. Thus, posts are indicated to retain the core material. Posts can be classified according to the fabrication technique into prefabricated (single visit appointment) or custom-made posts (two visit appointments). The prefabricated posts require cementing the post first then building up the core [5].

Nowadays, fiber-reinforced posts (FRPs) have emerged as a preferred choice for the rehabilitation of endodontically treated teeth. These posts offer several advantages over their metallic counterparts, including superior esthetic outcomes, as they can mimic the color and translucency of natural teeth. Additionally, FRPs possess a modulus of elasticity (18–40 GPa) close to that of dentin (18–20 GPa) in contrast to the metal post (190–200 GPa), reducing the risk of root fracture under occlusal stress [6, 7]. The ability to be bonded effectively with the dentinal walls through adhesive systems further enhances the retention and longevity of these restorations [8–10].

However, despite these advantages, the longevity of fiber-reinforced post restorations can be compromised by the failure of the bond between the post and the root canal dentin, leading to post debonding [11]. Bonding to dentin is very challenging due to the complexity of the tissue which varies in locations (radicular or coronal) and types (primary, secondary or tertiary) [12]. The failure of this bond is a result of a variety of factors, including improper cementation technique, impaired visibility, inadequate moisture control, or the failure to properly clean and prepare the root canal space before placing the post [13–15]. These factors highlight the complexity of achieving optimal bonding and retention in these restorations.

After root canal treatment and post space preparation, a smear layer is usually formed [16]. This layer consists of organic and inorganic debris that adhere to the dentin walls which prevents the cement from penetrating and adapting to the dentin and might influence the bond strength of the post [16]. In addition to the smear layer, there are endodontic sealer remnants that adhere to the dentinal wall that must be removed to expose the dentin [17]. In an in vitro study, remnants of endodontic sealers decreased the retention of the posts cemented with resin cement in comparison to canal obturated without a sealer [18].

To achieve durable bonding, the literature suggests various cleaning methods to remove the smear layer and sealer remnants. Sodium hypochlorite (NaOCl) is a commonly used irrigating solution during endodontic treatment [19]. This halogenated compound is known for its antimicrobial action and its ability to dissolve remaining pulp tissue and the organic components of uninstrumented dentin on the canal walls [20]. However, the effect of sodium hypochlorite on the bonding of restorative materials to dentin is a subject of debate [21]. Chlorhexidine gluconate, an antiseptic irrigant, is recommended as a less toxic alternative [22]. Furthermore, chlorhexidine gluconate can inhibit matrix metalloproteinases (MMPs), enzymes responsible for bond degradation [23]. Ethylene diamine tetra acetic acid (EDTA), a chelating agent, effectively removes the smear layer [24]. However, prolonged use of EDTA can lead to dentin erosion, which has a detrimental effect [25].

In recent years, novel cleaning agents such as Triethanolamine, polyethylene glycol,10-Methacryloyloxydecyl dihydrogen phosphate salt (Katana™ Cleaner, Kuraray Noritake Dental, Inc., Okayama, Japan) have been introduced. Katana™ Cleaner is a non-abrasive, universal dental cleaning agent specifically designed for various surfaces, including dentin, enamel, root canals, tooth abutments and indirect restorations [26]. This product has demonstrated promising results in enhancing the bonding performance of self-etch adhesives to ceramic restorations and is attracting interest for its potential applications in endodontics [27–29]. Air-borne particle abrasion has been employed on different surfaces to improve bonding [30]. However, the depth of the post space might limit the accessibility of air-borne particle abrasion to deeper regions. Therefore, this study aimed to evaluate the effect of different cleaning agents on the push-out bond strength of fiber-reinforced posts cemented with self-adhesive resin cement. Specifically, we sought to compare commonly used cleaning agents in clinical practice, such as phosphoric acid, sodium hypochlorite, air-borne particle abrasion and EDTA, with Katana™ Cleaner, a newly introduced cleaning agent. The null hypothesis posits that there is no significant difference in the push-out bond strength of fiber-reinforced posts cemented with self-adhesive resin cement after the application of different post space cleaning methods.

## Methods

### Experimental design

The sample size calculation was performed using G*Power 3.1.9.7 statistical software. The effect size f was set at 0.3, with a significance level of α = 0.05 and a power of 80%, across seven test groups. This calculation yielded a total required sample size of 140 specimens. We obtained two mid-root specimens per tooth, aiming for 20 specimens per group. To account for potential sampling or fabrication errors, the sample size was expanded to 26 specimens per group, which increased the power of the study to 92%.

### Ethical approval and teeth selection

The study was conducted in accordance with the Declaration of Helsinki and was approved by the Research Ethics Committee at King Abdulaziz University, Faculty of Dentistry, Protocol number 146-12-22 and approval date of 08/01/2023. Ninety-one single canal lower premolar teeth extracted for orthodontic purposes were selected from the Oral Surgery Clinics. All teeth were collected following informed consent and anonymized prior to use in the study. Teeth with crack lines, root fracture, caries, erosion and previous endodontic treatment were excluded. Teeth were collected for 3 months and stored in normal saline until the start of the study. All laboratory steps were done by three operators (K.A.A, A.A.A, H.A.A).

### Root canal preparations

The crowns were sectioned 1 mm above the cementoenamel junction with low-speed diamond saw (Allied techcut 4Low Speed Diamond Saw, Rancho Dominguez, CA USA) under water-coolant. Teeth were then endodontically treated using nickel-titanium (Ni-Ti) rotary files (ProTaper; Dentsply Maillefer, Ballaigues, Switzerland). The canals were enlarged to size F2 ProTaper file and 2 ml of 5.25% sodium hypochlorite (NaOCl; Chloraxid, Cerkamed, Poland) was used for irrigation. Root canals were then dried using paper points (Dentplus Absorbent paper points, Dentsply Sirona, NC USA) and obturated using gutta percha (Dentplus Gutta Percha Points, Dentsply Sirona, NC USA) and AH-26 sealer (Dentsply DeTrey, Konstanz, Germany). The specimens were kept in an incubator (Thermo ScientificTM HerathermTM, Thermo Fisher Scientific Inc., Waltham, MA, USA) at 37 °C with 100% humidity for seven days. Later, teeth were embedded in a clear acrylic resin (Idofast Orthodontica, Unidesa Odi, Madrid, Spain) for handling purposes.

### Post space preparation and experimental groups

Post spaces of 10 mm in length were prepared using a size 3 Gates Glidden drill (Gates Glidden drill, Produits Dentaires SA, Vevey, Switzerland), followed by a size 2 post drill from a fiber-reinforced post kit (Pillar Fiber Post kit, TiaDent, Iași, Romania). Post spaces were inspected visually using magnifying loupes at 5Х and periapical radiographs were taken to confirm the complete removal of gutta-percha and sealer remnants. The teeth were randomly divided into seven groups (13 teeth per group) according to the cleaning methods:

- Group 1: Control. Post spaces were irrigated with 2 ml of normal saline (Normal-Saline 0.9% W/V Infusion 500 ml, Pharmaceutical Solutions Industry, Saudi Arabia).
- Group 2: Sodium hypochlorite (NaOCl). Post spaces were irrigated with 2 ml of 5.25% NaOCl (Chloraxid, Cerkamed, Stalowa Wola, Poland).
- Group 3: Chlorhexidine (CHX). Post spaces were irrigated with 2 ml of 2% chlorhexidine (Consepsis, Ultradent, UT, USA).
- Group 4: Universal Cleaner. Katana™ Cleaner (Triethanolamine, polyethylene glycol,10-Methacryloyloxydecyl dihydrogen phosphate salt) (Kuraray Noritake Dental, Inc., Okayama, Japan) was rubbed into the post space using a microbrush for 10 s following the manufacturer’s instructions.
- Group 5: Air-borne particle abrasion. Post spaces were sandblasted (Duostar Z2, BEGO; Bremen, Germany) using 50 μm aluminum oxide particles (Korox50, BEGO, Bremen, Germany) under an air pressure of 2 bars for 10 s.
- Group 6: Chelating agent. Post spaces were irrigated with 2 ml of 17% ethylene diamine tetra acetic acid (EDTA, Pulpdent, Watertown, MA, USA) for 60 s.
- Group 7: Phosphoric acid. A 37% phosphoric acid etchant (FineEtch 37, Spident, Incheon, South Korea) was applied to the canal space using a microbrush for 15 s.

Following the cleaning procedure, all post spaces were rinsed with 2 ml of normal saline as a final wash and dried with paper points. Drying was confirmed when paper points were retrieved completely dry with no visible signs of moisture [31]. Automixing tips of the self-adhesive resin cement (RelyX^™^ U200 Automix, 3M ESPE, Seefeld, Germany) were inserted to the full working length and the post spaces were then filled with cement while the tip was withdrawn slowly, then the cement was also applied on size 2 fiber-reinforced posts (Pillar Fiber Post kit, TiaDent, Iași, Romania). The diameter of the post was 0.8 mm at the apical end and 1.6 mm at the coronal end, with a total taper of 8% (4.58°). The post was slowly inserted to the full working length. Posts were inserted with rotational torque < 0.5 N·cm and finger pressure < 1 N, standardized by a single operator. Pressure was maintained during setting to enhance cement adaptation and minimize void formation. The cementation step was completed by one operator to improve reproducibility. Light polymerization was performed from the coronal direction through the posts for 40 s using a Bluephase N MC Light Cure unit (Ivoclar Vivadent, Schaan, Liechtenstein) at 1200 mW/cm^2^. All specimens were then stored in normal saline in a 37 °C incubator for 48 h. The composition and manufacturers of the materials used in this study are summarized in Table 1.

**Table 1:** Compositions and manufacturers of the materials used in the current study

Material and Manufacturer	Description	Composition
**Chloraxid** Sodium Hypochlorite Cerkamed, Stalowa Wola, Poland	Irrigant and cleaner	5.25% of active chlorine, purified water
**Consepsis** Chlorhexidine Ultradent, UT, USA	Disinfectant and cleaner	Chlorhexidine gluconate, ethyl alcohol, polyethylene glycol, Dimethicone, oils, peppermint.
**Katana** ^™^ **Cleaner** Kuraray, Okayama, Japan	Universal cleaner	Triethanolamine, polyethylene glycol, 10-Methacryloyloxydecyl dihydrogen phosphate salt
**Korox50** BEGO, Bremen, Germany	Air-born particle abrasion	Aluminum oxide (Al_2_O_3_)
**EDTA** Pulpdent, Watertown, MA, USA	Chelation agent	Ethylene diamine tetra acetic acid
**FineEtch 37** Phosphoric acid Spident, Incheon, South Korea	Etchant	37% phosphoric acid in a proprietary gel matrix
**RelyX U200 Automix (RX)** 3M ESPE, Seefeld, Germany	Self adhesive resin cement.	Base: Methacrylate monomers containing phosphoric acid groups, methacrylate monomers, silanated fillers, initiator components, stabilizer, Rheological additives. Catalyst: Methacrylate monomers, alkaline (basic) fillers, silanated fillers, initiator components, stabilizer, pigments.

### Specimen preparation and mechanical testing

Specimens were sectioned to obtain two mid-root slices, each was 2 mm thick using a precision low-speed diamond saw. All slices were obtained from the middle third of the root, where dentinal tubules tend to be more consistent and predominantly radial in orientation [32]. This standardization was intended to minimize variability due to tubule direction. Each slice was treated as an independent specimen (*n* = 26 per group). This slice-based approach is consistent with recent studies where root slices were analyzed independently in push-out bond strength testing [33–35]. The coronal surfaces were marked to be identified during testing. All the specimens meeting the inclusion criteria (intact dentin, no cracks or defects) were included. No randomization was applied beyond this point to ensure uniformity across specimens. All specimens were then subjected to an artificial aging process, thermocycling at 5–55 °C for 5000 cycles (simulate approximatly 6 months clinical function) in the SD Mechatronik Thermocycler (JulaBO GmbH; Seelbach, Germany) [36]. Each cycle took 70 s (30 s dwell time for each bath and 10 s transfer time). The Push-out bond strength test was conducted using a universal testing machine (INSTRON, Model #5944, load cell 2 KN) from the apical to coronal direction (marked coronal surface was placed downward) at 0.5 mm/minute rate until the post was dislodged. The maximum load to failure was measured in newtons (N) and then converted to bond strength (MPa) by dividing the load by the surface area of the bonding area. The bonding surface area was calculated using the following formula:

$$\:A=\:\pi\:\left(R\:+\:r\right)\sqrt{\left\{{h}^{2}+\:{\left(R\:-\:r\right)}^{2}\right\}}$$

**Fig. 1:**
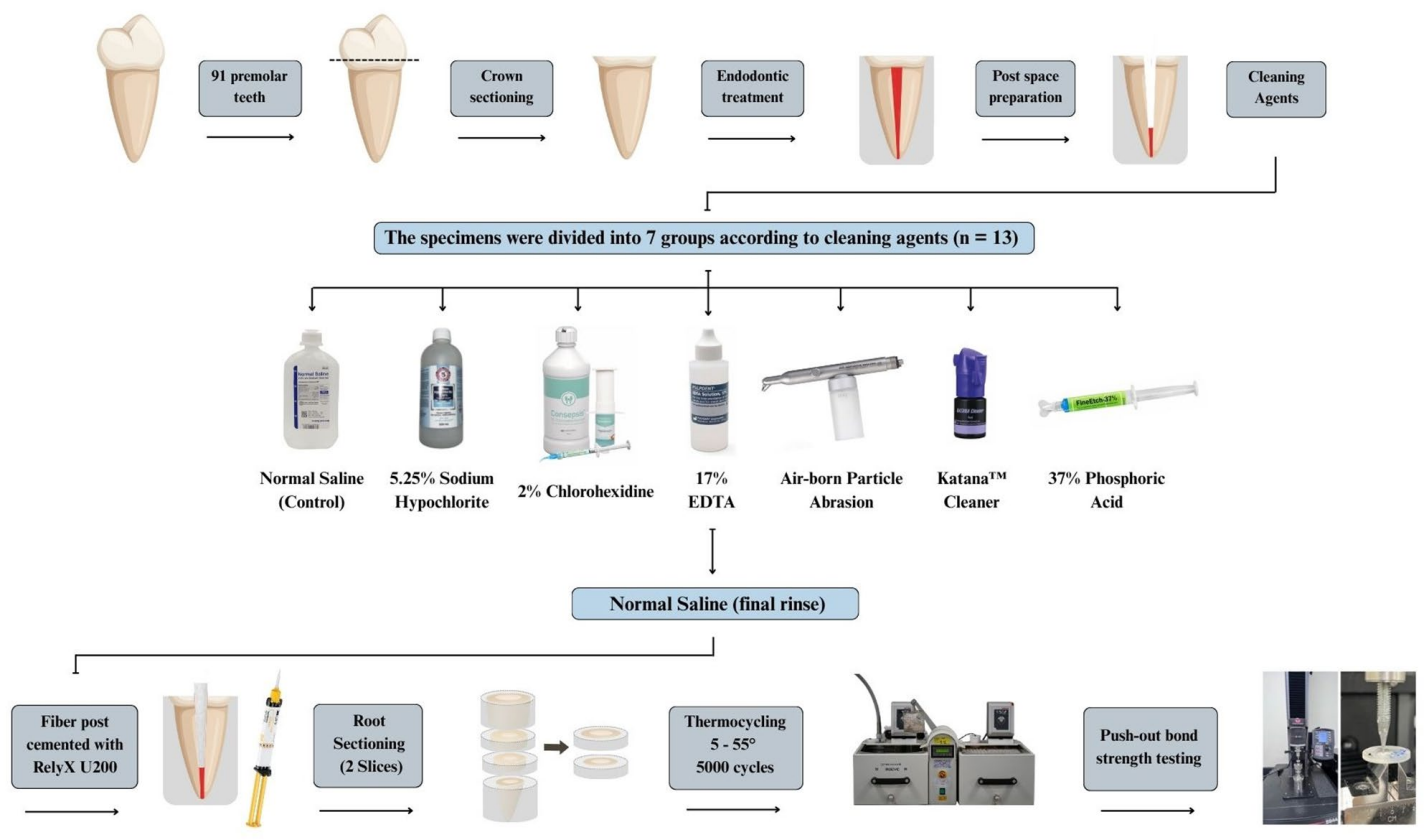
Flowchart of the study design

Where R represents the coronal radius of the post, r denotes the apical radius of the post and h is the thickness of the slice [37]. Figure 1 summaries the study design.

### Failure mode analysis

After the push-out bond strength test was completed, every specimen was inspected under a light stereomicroscope (MX 7520, Meiji Techno, Hicksville, New York, USA) at 40Х magnification. Three modes of failure were identified: (1) adhesive failure between the cement and post where the root surface is completely covered with cement, (2) adhesive failure between the cement and root, where the root surface is completely exposed with no cement, or (3) mixed failure with remnants of the cement covering some parts of the root. Percentages were calculated by dividing the number of slices with a given failure mode by the total number of slices per group (*n* = 26).

### Statistical analysis

Statistical analysis was completed using JMP 17 Statistical Discovery from SAS software (SAS Campus Drive. Cary, NC, USA). Shapiro–Wilk’s test of normality was used to evaluate the normality of the push-out bond strength test data, and it was found they did not follow a normal distribution. Kruskal-Wallis test (non-parametric testing) was used to evaluate the push-out bond strength of fiber-reinforced post cemented with self-adhesive resin cement after using different post space cleaning methods, followed by nonparametric comparisons with control using Dunn Method. Chi-square test was used to evaluate the failure mode distribution. All statistical tests were performed at significance level α = 0.05.

## Results

**Fig. 2:**
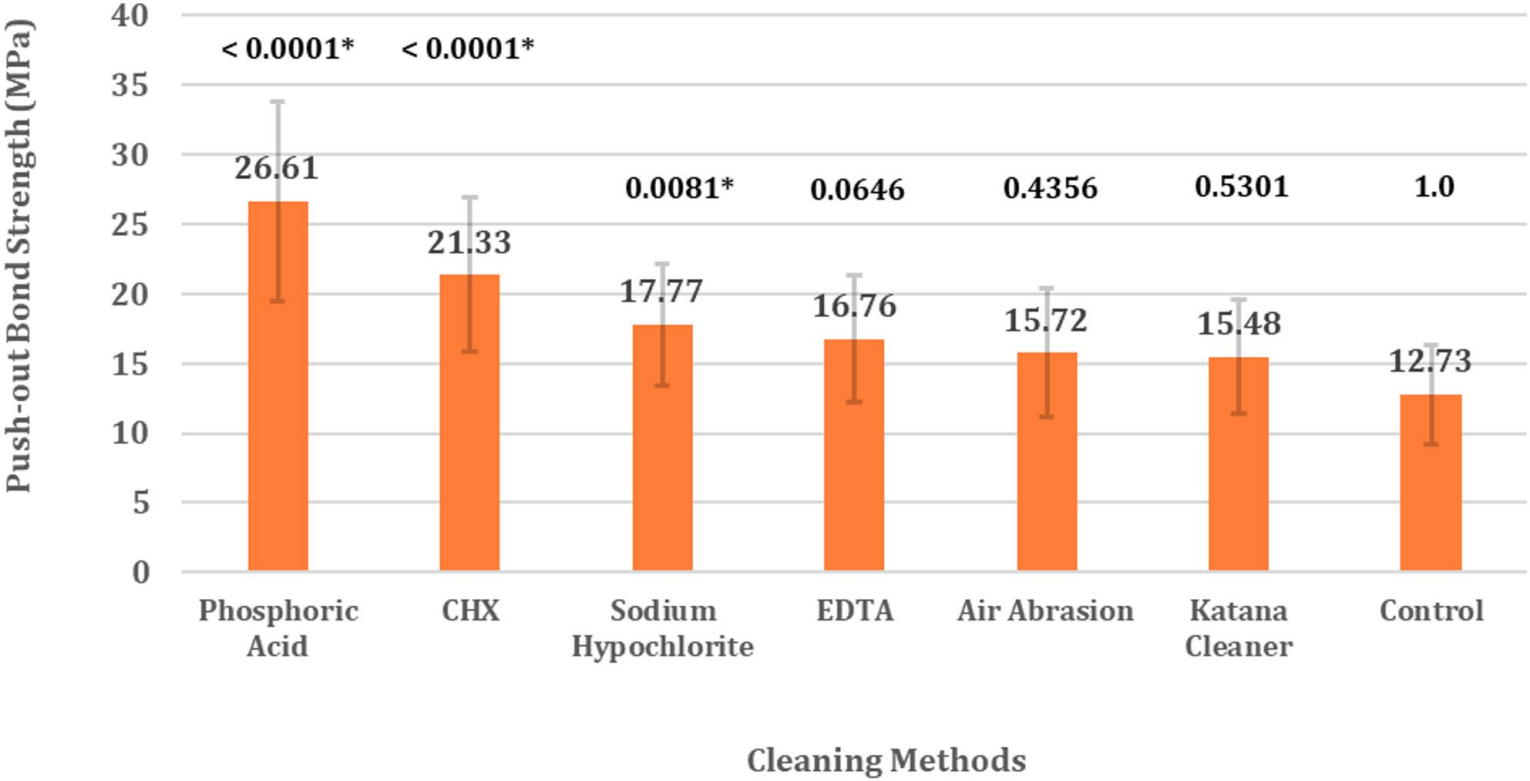
Push-out bond strength of fiber-reinforced post to root dentin after using different cleaning agents, ranked from highest to lowest. Nonparametric Comparisons with Control Group Using Dunn Method, *Significant at *p* < 0.05

The mean push-out bond strength values and standard deviations (SD) of fiber-reinforced posts to root dentin for the experimental and control groups are summarized in Table 2.. The Kruskal-Wallis test revealed a significant difference in PBS between the test groups (*p* < 0.0001). Nonparametric comparisons of the PBS of different groups with control using Dunn Method revealed that the PBS of group treated with phosphoric acid etching (26.61 ± 7.22 MPa), CHX (21.33 ± 5.54 MPa) and Sodium Hypochlorite (17.77 ± 4.37 MPa) demonstrated significantly higher PBS than the control group. Regarding the PBS of the remaining groups; EDTA (16.76 ± 4.54 MPa), air-borne particle abrasion (15.72 ± 4.61 MPa), and Katana™ Cleaner (15.48 ± 4.06 MPa) were not statistically different from control group. (Fig. 2).

**Table 2.** Descriptive data and statistical analysis of push-out bond strength, ranked from highest to lowest

Group (Cleaning Agent)	Mean ± SD (MPa)	95% Confidence Interval		Kruskal-Wallis Test *p-*value	Nonparametric Comparisons with Control Group Using Dunn Method	
		Lower 95%	Upper 95%		Score Mean Difference	*p*-value
Phosphoric Acid	26.61 ± 7.22	23.56	29.65	< 0.0001*	91.28	< 0.0001*
CHX	21.33 ± 5.54	18.74	23.92		69.6	< 0.0001*
Sodium Hypochlorite	17.77 ± 4.37	15.93	19.62		44.1	0.0081*
EDTA	16.76 ± 4.54	14.89	18.64		34.76	0.0646
air-born particle abrasion	15.72 ± 4.61	13.62	17.81		24.7	0.4356
Katana™ Cleaner	15.48 ± 4.06	13.76	17.20		24.21	0.5301
Control	12.73 ± 3.53	11.12	14.33		0.0	1.0

*Significant at *p* < 0.05

**Fig. 3:**
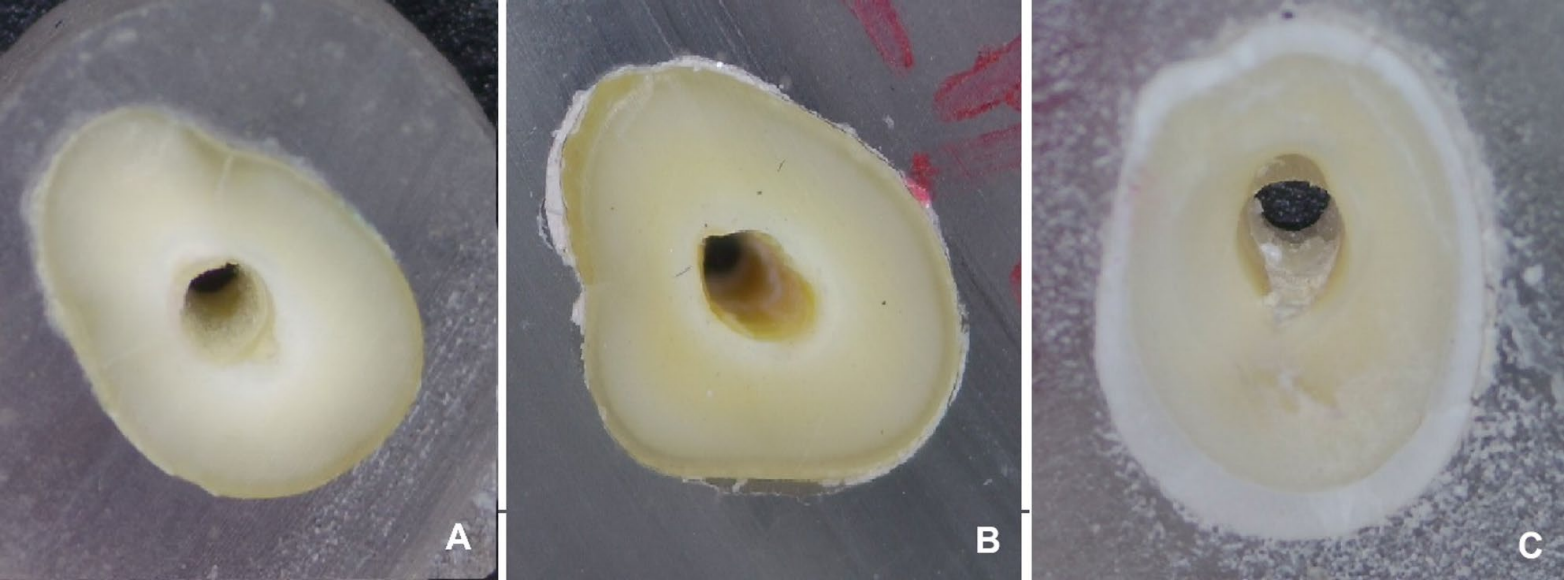
Stereomicroscopic images of different Failure modes at 40Х. **A** Failure mode 1: adhesive failure between cement and post (root surface is completely covered with cement). **B** Failure mode 2: adhesive failure between cement and root (root surface is completely exposed with no cement). **C** Failure mode 3: mixed failure (remnant of cement covering some part of root)

For failure mode evaluation, Chi-square test showed there is a significant difference in the failure mode distribution between test groups (*p* = 0.0005) (Table 3). Most specimens in phosphoric acid (80%), chlorhexidine (90%) and EDTA (80%) groups had failed by failure mode 1, where the root surface is completely covered with cement. Meanwhile, more failure mode 3 (mixed failures) were found with air-born particle abrasion (70%), control group (55.56%) and Katana^™^ Cleaner (50%) groups. A less distinct pattern was reported with sodium hypochlorite, 40% for failure modes 1 and 2, and only 20% for failure mode 3 (Fig. 3).

**Table 3:** Chi-square test, effect of different cleaning agents on the failure mode

Failure Modes	Phosphoric Acid	CHX	Sodium Hypochlorite	EDTA	Air-born particle abrasion	Katana^™^ Cleaner	Control	Chi-square test	*p*- Value
1	80%	90%	40%	80%	0%	20%	33.33%	35.006	0.0005*
2	20%	0%	40%	0%	30%	30%	11.11%		
3	0%	10%	20%	20%	70%	50%	55.56%		

Failure modes: Percentages calculated per slice

(1) adhesive failure between cement and post (root surface is completely covered with cement)

(2) adhesive failure between cement and root (root surface is completely exposed with no cement)

(3) mixed failure (remnant of cement covering some part of root)

*Significant at *p* < 0.05

## Discussion

The bonding of a post to dentin is crucial for the long-term success of indirect restorations. This study investigated the impact of various cleaning agents on the push-out bond strength of fiber-reinforced posts cemented with self-adhesive resin cement to radicular dentin. The post space cleaning methods employed before fiber-reinforced post cementation significantly influenced the push-out bond strength (*p* < 0.0001), thus leading to the rejection of the null hypothesis. These findings align with previous research highlighting the importance of cleaning protocols in determining bond strength values [13, 38, 39].

Fiber-reinforced posts are commonly used in endodontically treated teeth. The bond strength between the fiber post and the root dentin is influenced by several factors, including the type of cement used and the cleaning method of the root canal [40, 41]. Prefabricated fiber-reinforced posts were chosen for this study due to their lower modulus of elasticity, which facilitates a more even distribution of stress across the radicular dentin. A tapered post design was selected to ensure a uniform, thin layer of cement between the post and the dentin along the entire post space [41, 42]. In this study, push-out bond strength test was used to evaluate the retention of the prefabricated fiber-reinforced posts. It is considered a suitable method because it offers a precise evaluation of the bonding process, is easy to use, provides comparison data among the groups and mimics clinical scenarios [43–45].

The cement system used for post cementation is critical for durability. Resin cements are classified into three types based on their etching mode: etch-and-rinse, self-etch, and self-adhesive [46]. Due to its convenience and speed, self-adhesive resin cement is most commonly used in clinical practice. However, incorporating an etching step with self-adhesive resin cement significantly increased the bond strength (26.61 ± 7.22 MPa) compared to the control group (12.73 ± 3.53 MPa). Adding the etching step to self-adhesive resin cement is an optional step, not mandatory as conventional (total etch) resin cement. This promotes better micromechanical retention for resin tags and improves cement penetration. Jalali et al., used self-etch resin cement to cement fiber posts and found that etching the canal showed the significant increase in bond strength [13]. Etching dentin removes the smear layer, creates microporosities and enhances surface wettability, resulting in improved resin tag formation within the dentinal tubules [33].

In the present study, three irrigation materials were used in this study, Chlorhexidine, Sodium hypochlorite and Saline as the control. Chlorhexidine resulted in the second highest bond strength amongst all groups (21.33 ± 5.54 MPa). Chlorhexidine is a well-known antimicrobial solution with broad-spectrum activity [23]. Good PBS might be attributed to its ability to inhibit proteolytic enzymes that degrade collagen fibers, improving the bond durability [12]. Sodium hypochlorite is the most widely used irrigating solution in endodontics due to its ability to dissolve pulp tissue and the organic components of smear layer from the canal walls. The use of Sodium hypochlorite to clean post space before cementation of fiber post yielded bond strength of (17.77 ± 4.37 MPa), which was also significantly higher when compared to the saline control group (12.73 ± 3.53 MPa). However, there is some concern regarding irrigation with sodium hypochlorite. It can create an oxygen-rich layer on the dentin surface, which can inhibit resin polymerization and consequently reduce bond strength [47].

The last two materials used were EDTA and Katana™ Cleaner. The use of EDTA as a cleaning agent to achieve an etching effect in the post space resulted in bond strength values (16.76 ± 4.54 MPa) that were statistically similar to those observed with Katana™ Cleaner and air-borne particle abrasion. However, the literature suggests that prolonged application of EDTA can lead to dentin erosion [12]. EDTA, a chelating agent with the capacity to demineralize the surface, may require longer application time to effectively dissolve the smear layer and penetrate deeper into the dentin surface [48]. Katana™ Cleaner was introduced as a cleaning agent for both dental hard tissues and restorative materials [26, 27, 45]. It is designed for extraoral and intraoral cleaning applications, including restorative and endodontic procedures [46, 47]. However, its effectiveness in cleaning post spaces was not promising, as it did not significantly improve bonding strength (15.48 ± 4.06 MPa). Alzahrani et al. [48] reported similar findings when Katana™ Cleaner was used to clean dentin surfaces contaminated with a hemostatic agent. They attributed the lower bond strength to the material’s low acidity, which may promote the activation of matrix metalloproteinases and subsequent degradation of the hybrid layer in the presence of moisture. Similarly, air-borne particle abrasion did not result in a significant increase in bond strength (15.72 ± 4.61 MPa), possibly due to the limited accessibility of abrasive particles to the deeper regions of the post space.

The failure mode evaluation provided further insights into bonding effectiveness. The most favorable failure mode was characterized by the dentin surface being completely covered with cement, indicating strong adhesion of the cement to the root surface (Failure Mode 1). This suggests that the cleaning agent was effective in dissolving the smear layer and facilitating adequate bonding. In contrast, mixed failures (Failure Mode 3), in which remnants of cement covered only part of the dentin surface, suggest a less effective cleaning protocol. This mode reflects suboptimal smear layer removal and inadequate surface wettability, resulting in moderate bonding quality. The least favorable mode (Failure Mode 2) was observed when the dentin surface remained clean with no cement remnants, indicating that bonding occurred primarily to the post surface and not to the root dentin. A higher frequency of favorable failure mode (Failure Mode 1) was seen in groups treated with the most effective cleaning agents—phosphoric acid (80%), chlorhexidine (90%), and EDTA (80%)—all of which also demonstrated higher push-out bond strengths.

The clinical relevance of the present study highlights the importance of thoroughly cleaning the post space prior to fiber post cementation, as this step significantly influences the durability of the final restoration. Among the methods evaluated, acid etching with phosphoric acid proved to be the simplest and most effective technique for clinicians.

The comparative analysis of various cleaning agents under thermocycling conditions aimed to simulate the intraoral environment and provide more clinically applicable results. However, the in vitro nature of this study remains a limitation. Another limitation lies in the specimen configuration; incorporating a full restoration system—including crown, core, and post—and subjecting it to cyclic mechanical loading would better reflect clinical conditions. Despite these limitations, the push-out bond strength test remains a widely accepted and reliable method for evaluating post retention and ranking bonding performance of different treatments. These findings also underscore the need for further research. Future investigations should include scanning electron microscopy to assess the dentin surface morphology post-cleaning and energy-dispersive spectroscopy to analyze the chemical composition of debonded surfaces. Additionally, exploring the influence of different resin cement systems, including etch-and-rinse or self-etch adhesives, may yield different outcomes and should be further examined.

## Conclusions

Adhesion to intraradicular dentin remains a challenging aspect of restorative dentistry. This study highlights the significant impact of cleaning agents on the push-out bond strength of fiber-reinforced posts to root dentin. Among the tested agents, phosphoric acid demonstrated the highest bond strength, followed by chlorhexidine and sodium hypochlorite. While other cleaning agents such as EDTA, and Katana™ Cleaner (Triethanolamine, polyethylene glycol,10-Methacryloyloxydecyl dihydrogen phosphate salt) were not significantly different from the control group. The simplest and most effective method for clinicians is acid etching the canal with 37% phosphoric acid.

## Supplementary Information

Supplementary Material 1.

Supplementary Material 2.

## Data Availability

The data that support the findings of this study are available upon reasonable request from the corresponding author.

## Abbreviations

FRPs: Fiber reinforced posts
PBS: Push-out bond strength
NaOCl: Sodium hypochlorite
EDTA: Ethylene diamine tetra acetic acid
CHX: Chlorhexidine gluconate
SD: Standard deviations
