# Supplementary material for: Does cleaning of post space before cementation of fiber reinforced post affect the push-out bond strength to resin cement?
Source: BMC Oral Health. 2025 Dec 22;25:1947. doi: 10.1186/s12903-025-07483-0 (PMC12746632; doi:10.1186/s12903-025-07483-0)
Supplement: Supplementary file 1 — Supplementary Material 1. [file 12903_2025_7483_MOESM1_ESM.docx]

**Does Cleaning of Post Space Before Cementation of Fiber Reinforced Post Affect the Push-Out Bond Strength to Resin Cement?**

Maher S. Hajjaj^1^*, Khalid A. Alghamdi^2^, Abdulrahman A. Alshehri^3^, Hassan A. Almusallam^4^, Nabeel M. Munshi^5^, Osamah A. Alsulimani^6^, Naseeba H. Khouja^1^, Yousef A. Alnowailaty^1^ and Saeed J. Alzahrani^1^

1. Department of Restorative Dentistry, Faculty of Dentistry, King Abdulaziz University, Jeddah, Saudi Arabia; [mhajjaj@kau.edu.sa](mailto:mhajjaj@kau.edu.sa) (M.S.H.), [nkhooja@kau.edu.sa](mailto:nkhooja@kau.edu.sa) (N.H.K.), [yalnowailaty@kau.edu.sa](mailto:yalnowailaty@kau.edu.sa) (Y.A.A), [sjalzahrani1@kau.edu.sa](mailto:sjalzahrani1@kau.edu.sa) (S.J.A.)
2. Family Dentistry Department, Riyadh Elm University, Riyadh, Saudi Arabia; [khalid.alghamdi2024@student.riyadh.edu.sa](mailto:khalid.alghamdi2024@student.riyadh.edu.sa) (K.A.A.).
3. General Dentist, Private Practice, Jeddah, Saudi Arabia; [aalshehri22@hotmail.com](mailto:aalshehri22@hotmail.com) (A.A.A.)
4. Clinical Instructor, Faculty of Dentistry, King Faisal University, Hufof, Saudi Arabia; [haalmusallam@kfu.edu.sa](mailto:haalmusallam@kfu.edu.sa) (H.A.A.)
5. Department of Oral and Maxillofacial Prosthodontics, Faculty of Dentistry, King Abdulaziz University, Jeddah, Saudi Arabia; [nmunshi@kau.edu.sa](mailto:nmunshi@kau.edu.sa) (N.M.M.)
6. Department of Oral Diagnostic Sciences, Faculty of Dentistry, King Abdulaziz University, Jeddah, Saudi Arabia; [oaalsulimani@kau.edu.sa](mailto:oaalsulimani@kau.edu.sa) (O.A.A.)

Correspondence: Maher S. Hajjaj, BDS, MSD, CAGS, FRCDC, DScD, Department of Restorative Dentistry, Faculty of Dentistry, King Abdulaziz University, Jeddah, Saudi Arabia, P.O. Box 80209 Jeddah, 21589, Saudi Arabia.

Email: [mhajjaj@kau.edu.sa](mailto:mhajjaj@kau.edu.sa)

+966504637871

**Acknowledgments:**

The authors would like to thank the Advanced Technology Dental Research Laboratory at Faculty of Dentistry, King Abdulaziz University for the help with the laboratory work done on this project.

**Ethics approval and consent to participate:** The study was conducted in accordance with the Declaration of Helsinki and was approved by the Research Ethics Committee at King Abdulaziz University, Faculty of Dentistry, Protocol number 146-12-22 and approval date of 08/01/2023.

**Consent for publication:** Not Applicable.

**Competing interests:** The authors declare no conflicts of interest and do not have any financial interest in companies whose materials are included in this article.

**Disclosure:** This article is a revised and expanded version of a paper titled “Does cleaning of post space before cementation of fiber-reinforced post affect push-out bond strength to resin cement?" which was presented at the 36th Saudi International Dental Conference, Riyadh, Saudi Arabia on 16-18th of January 2025 and 16th Annual Research and Innovation Day, Riyadh Elm University, Riyadh, Saudi Arabia on 16th of April 2025.
