# Supplementary figures and images for: Does cleaning of post space before cementation of fiber reinforced post affect the push-out bond strength to resin cement?
Source: BMC Oral Health. 2025 Dec 22;25:1947. doi: 10.1186/s12903-025-07483-0 (PMC12746632; doi:10.1186/s12903-025-07483-0)

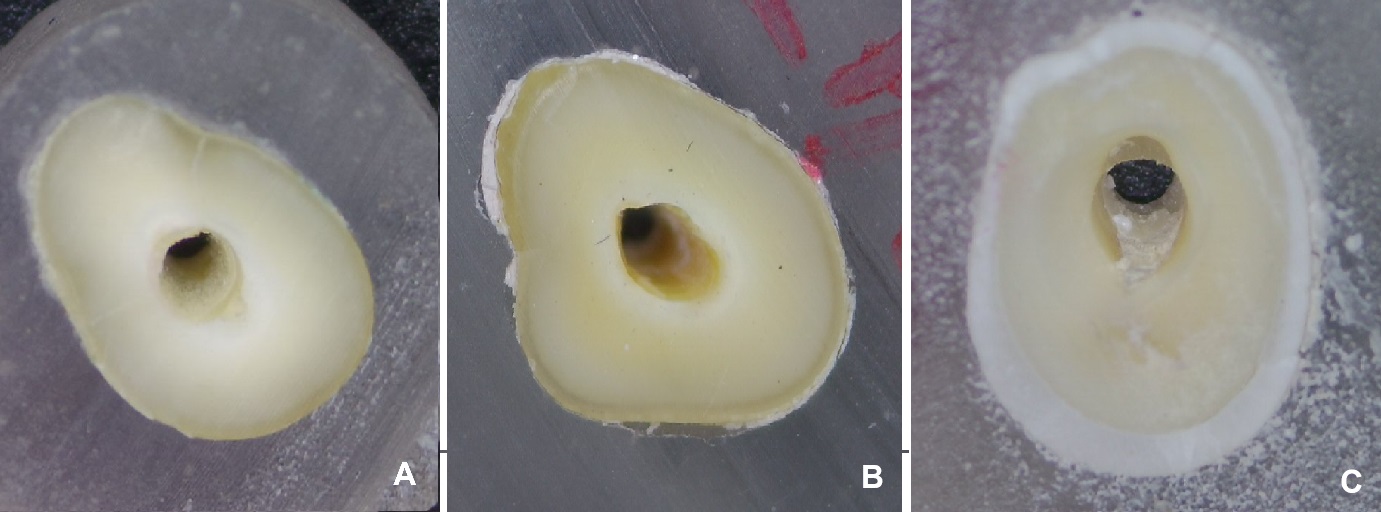

Supplement: Supplementary file 2 — Supplementary Material 2. [file 12903_2025_7483_MOESM2_ESM.jpg]
